# Deep learning for accurate tumour volume measurement and prediction of therapy response in paediatric osteosarcoma

**DOI:** 10.1007/s00330-025-12115-w

**Published:** 2025-11-01

**Authors:** Ricarda von Krüchten, Michael Barrow, Lisa Adams, Shashi Bhushan Singh, Zahra Shokri Varniab, Vidyani Suryadevara, Prinska Ghimire, Allison Pribnow, Jing Qi, Dylan Applin, Yashas Ullas Lokesha, Kerem Nernekli, Heike E. Daldrup-Link

**Affiliations:** 1 Department of Radiology, Molecular Imaging Program at Stanford (MIPS), Stanford University School of Medicine, Stanford, CA USA; 2 Department of Pediatrics, Stanford University School of Medicine, Stanford, CA USA; 3 Department of Radiology, Children’s Wisconsin, The Medical College of Wisconsin, Milwaukee, WI USA

**Keywords:** Magnetic resonance imaging, Paediatric osteosarcoma, Convolutional neural networks, Tumour volume segmentation, Therapy response prediction

## Abstract

**Objectives:**

To assess treatment response in osteosarcoma, two automated convolutional neural networks (CNNs) were developed to quantify tumour volumes and predict response to induction chemotherapy using histopathology as the reference standard.

**Materials and methods:**

This retrospective, multicentre study included magnetic resonance imaging (MRI) scans from osteosarcoma patients acquired between January 2006 and July 2024. A 3D U-Net CNN segmented tumours and calculated volumes at baseline and post-chemotherapy. A second CNN predicted treatment response based on MRI-derived tumour volume changes using histopathologic necrosis (≥ 90%) as the reference standard. Both models were trained on 162 scans from 81 patients (Centre A) and validated on 40 scans from 20 patients (10 per centre) with Centre B as the external test set. Human readers measured 3D tumour diameters and volumes, compared with CNN-derived volumes using Spearman’s correlation, Bland–Altman plots, and Dice coefficients. Prediction performance was assessed using accuracy, sensitivity, and specificity, with significance determined by agreement metrics.

**Results:**

Patients from Centre A had a mean age of 15 ± 5 years (52 males), and from Centre B a mean age of 13 ± 0 years (8 males). CNN- and human-derived tumour volumes showed strong correlation (Centre A: *r* = 0.98, Centre B: *r* = 0.95; *p* < 0.001). Dice coefficients were 0.86 (Centre A) and 0.81 (Centre B), with median Hausdorff distances of 15.0 mm and 14.2 mm. The response prediction model classified 16/20 cases (80% accuracy) with 90% sensitivity and 70% specificity.

**Conclusion:**

CNN-derived tumour volume measurements were comparable to human assessments. CNN-based volume changes predicted histopathologic response to chemotherapy in paediatric osteosarcoma.

**Key Points:**

***Question*** *Accurate, noninvasive assessment of treatment response in paediatric osteosarcoma is limited by its reliance on manual tumour measurements and post-surgical histopathology.*

***Findings*** *Automated deep learning accurately measured tumour volumes on MRI and predicted chemotherapy response with 80% accuracy, 90% sensitivity, and 70% specificity.*

***Clinical relevance*** *Automated deep learning enables accurate tumour volume assessment and prediction of chemotherapy response in paediatric osteosarcoma, offering a noninvasive tool to support and refine patient management*.

**Graphical Abstract:**

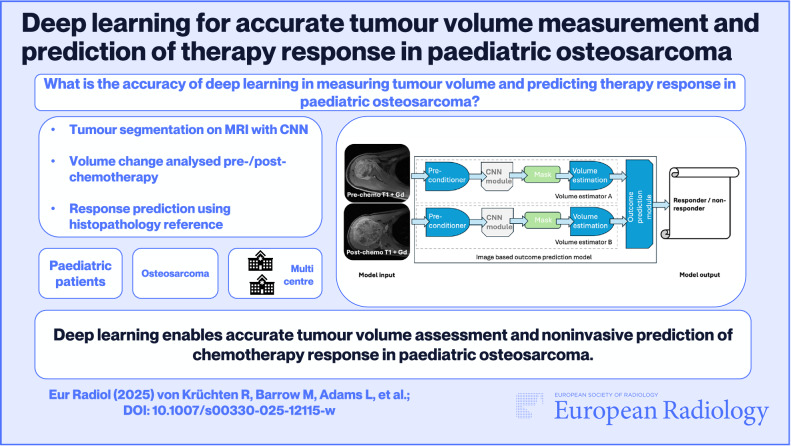

## Introduction

Osteosarcoma is the most common primary malignant bone tumour in children and young adults. The current standard of care involves neoadjuvant combination chemotherapy with high-dose methotrexate, adriamycin/doxorubicin, cisplatin (MAP), followed by surgical resection and adjuvant chemotherapy [1, 2]. Despite significant advances in multi-agent chemotherapy since the 1980s, progression-free survival rates have remained largely unchanged over the past 20 to 30 years, with 5-year event-free survival rates for localised disease typically ranging between 60% and 70% and only 30% for metastatic disease [3]. While the presence of metastatic disease is the strongest predictor of survival [4], histological response of the primary tumour to chemotherapy has been described as another important predictor of survival [5]. This threshold of ≥ 90% tumour necrosis indicates favourable histologic response to chemotherapy and has been widely adopted as a benchmark for evaluating treatment efficacy and used for risk stratification in clinical trials [6, 7]. However, the utility of histological tumour response as a prognostic indicator in osteosarcoma remains a subject of ongoing debate. A recent study suggested that the prognostic value of tumour necrosis may be affected by the intensity of induction chemotherapy [4].

MRI-based tumour volume changes have been proposed as a non-invasive biomarker to fill this role. Early research demonstrated a correlation between MRI-derived three-dimensional volume measurements and histopathologic response, suggesting a potential role in treatment evaluation [8]. However, the methods commonly used in clinical practice, such as three‑diameter measurement and estimating volume with the ellipsoid formula, lack reproducibility and suffer from high inter-observer variability, limiting their reliability [9].

Manual MRI tumour segmentation is time-consuming and impractical for routine practice. In recent years, deep learning-based convolutional neural networks (CNNs) have shown considerable promise for automated tumour segmentation across a variety of neoplasms, including benign tumours such as meningiomas [10] and malignancies such as non-small cell lung cancer [11]. Despite these advances, their use in paediatric oncology—particularly in osteosarcomas—remains largely unexplored. Although artificial intelligence (AI) is increasingly applied in oncologic imaging, studies focusing on paediatric radiology are scarce. Given the rarity of osteosarcoma and the challenges of conducting large-scale AI studies in children, robust, validated AI models are urgently needed for this vulnerable population.

To address this gap, we developed a fully automated CNN for tumour volume measurement in osteosarcoma. This study aimed to evaluate its accuracy compared to human readers and its ability to differentiate responders from non-responders based on tumour volume changes following induction chemotherapy.

## Materials and methods

### Patients

**Fig. 1:**
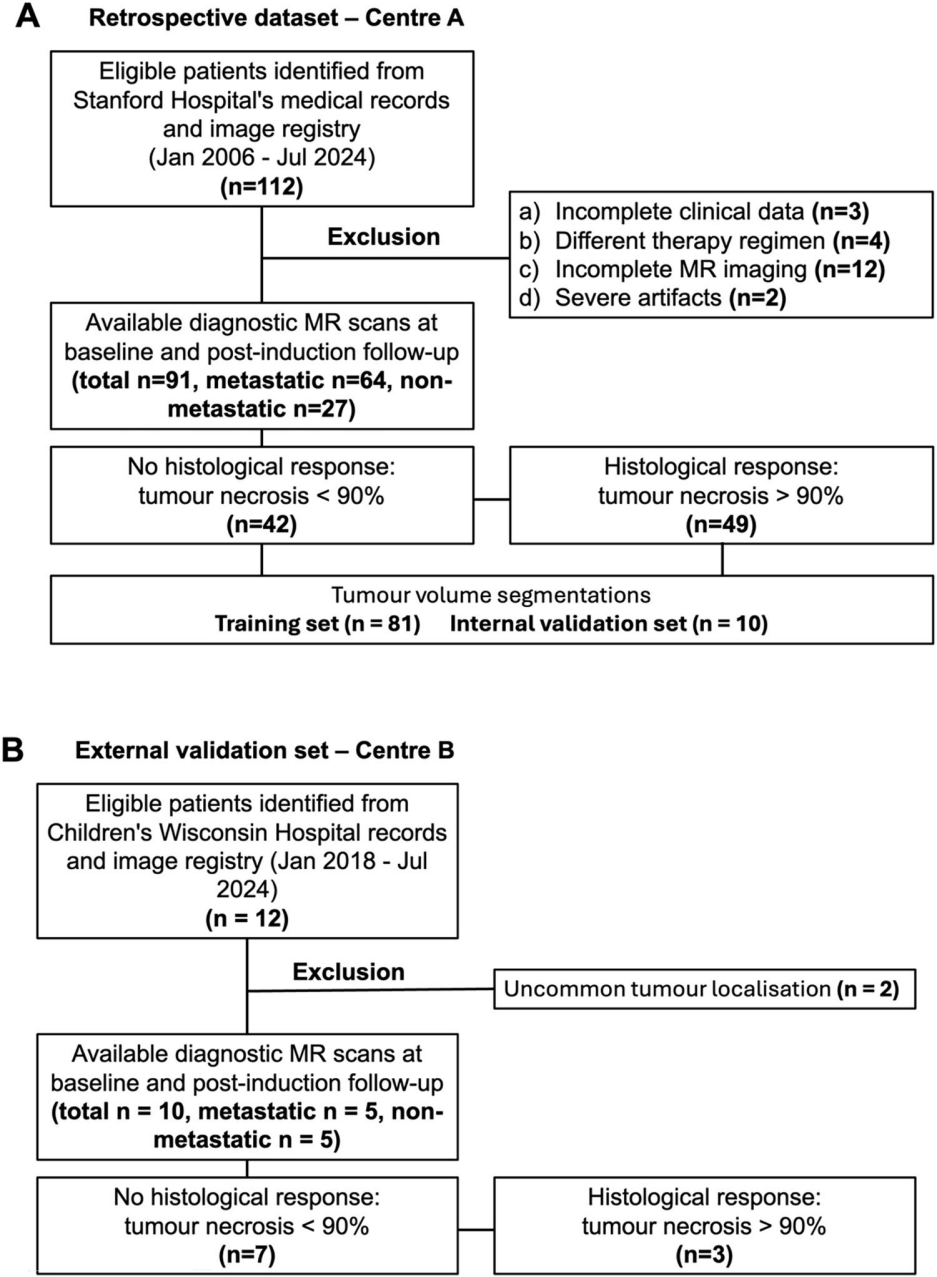
Flowchart of the study. **A** The training and internal validation set consisted of retrospective data collected from patients treated at Centre A. **B** The external validation set was derived from a separate cohort of patients treated at Centre B

This multicentre, retrospective study was approved by the institutional review board (IRB 48854), with patient consent waived due to its retrospective design. At Centre A (Stanford University), we identified 112 patients (< 26 years) diagnosed with biopsy-proven high-grade osteosarcoma between January 2006 and July 2024. Inclusion criteria were the availability of MRI scans at baseline and after MAP chemotherapy performed within 4 months. Exclusions (*n* = 21) included missing histopathological necrosis reports (*n* = 3), tumour surgery before chemotherapy or > 4 weeks after the second MRI (*n* = 4), incomplete MRI scans (*n* = 12), and severe artifacts (*n* = 2) (Fig. 1). The final cohort comprised 91 patients (39 females, 52 males, aged 6–26 years, mean 15 ± 5 years) (Table 1). The training set included 81 patients (34 females, 47 males, age range 5–25 years, mean 15 ± 5 years) with 162 annotated MRI scans (pre- and post-chemotherapy). The internal validation set consisted of 10 patients (5 females, 5 males, age 9–26 years, mean 16 ± 5 years), each with two MRI scans (pre- and post-chemotherapy) (Fig. 1A). Our CNN model underwent external validation using 20 MRI scans from 10 patients at Centre B (Children’s Wisconsin; 2 females, 8 males, aged 6–18 years, mean 13 ± 0 years) (Table 1). MRI data from Centre B were collected between January 2018 and July 2024 and met the same inclusion criteria as Centre A (Fig. 1B).

**Table 1:** Demographics and clinical findings in patients with osteosarcoma in two centres

	Centre A (*n* = 91)	Centre B (*n* = 10)
Sex		
Female	39 (43%)	2 (20%)
Male	52 (57%)	8 (80%)
Age at diagnosis, years		
	15 (6; 26)	14 (6; 18)
Ethnicity		
Hispanic or Latino	32 (35%)	0
Not Hispanic or Latino	59 (65%)	10 (100%)
American Indian	0	0
Alaska native	0	0
Native Hawaiian or other Pacific islander	0	0
Race		
White	50 (55%)	8 (80%)
Black	7 (8%)	2 (20%)
Asian	34 (37%)	0
Primary tumour site		
Femur	46 (51%)	2 (20%)
Tibia	18 (20%)	7 (70%)
Fibula	5 (5%)	0
Humerus	8 (8%)	1 (10%)
Radius	3 (3%)	0
Other	11 (12%)	0
Histological response		
≥ 90%	49 (54%)	3 (30%)
< 90%	42 (46%)	7 (70%)
Metastases		
	64 (57%)	4 (40%)
Survival (years)		
≥ 5	14 (15%)	2 (66%)
< 5	63 (69%)	1 (33%)
Recurrence (< 5 years)		
Yes	44 (48%)	0 (0%)
No	47 (52%)	10 (100%)

Centre A (*n* = 91; comprising training and internal validation data) and Centre B (*n* = 10) for external validation

The values represent the median (minimum, maximum) for continuous variables or frequency along with percentage (%) for categorical variables

Clinical characteristics between the training and external validation sets were well-balanced (Table 1). All patients received neoadjuvant MAP chemotherapy over 9 ± 3 weeks. Post-chemotherapy MRI scans were performed 10 ± 4 weeks after baseline, with local control surgery at 15 ± 10 weeks.

At Centre A, MRI scans for 90 patients were acquired at baseline and post-chemotherapy using 3 T Discovery MR750, 3 T Signa HDxT, or 3 T GE SIGNA™ PET-MRI AIR™ scanners. At Centre B, patients underwent MRI on 1.5 T Philips Ingenia, 1.5 T Siemens Aera, Avanto, Magnetom Symphony, 3 T Magnetom Skyra, or 3 T GE SIGNA™ Champion PET-MRI scanners.

The imaging protocol included axial, coronal, and sagittal T1-weighted sequences, axial and sagittal fat-saturated T2-FSE sequences, axial DW images (*b*-values: 50 and 600/800 s/mm²), and fat-saturated T1 sequences acquired after 0.1 mmol/kg Gadolinium (Gd) injection (Gadobutrol, Gadavist®, Bayer HealthCare Pharmaceuticals). Different T1-weighted sequences were used both within and between centres, reflecting variations in scanner models and institutional protocols.

### Manual tumour measurements and annotation

Two board-certified radiologists (reader 1 and reader 2), each with a two-year subspecialty focus in oncologic imaging—including one year of dedicated experience in paediatric cancer imaging with a particular emphasis on bone tumours—performed all tumour measurements and annotations. Reader 1 manually annotated the primary tumour on Gd-enhanced T1-weighted MR images using 3D Slicer (http://www.slicer.org) [12]. Segmentation was performed in the axial plane. To improve spatial alignment and accuracy, Gd-enhanced images were superimposed with plain T1 and T2-weighted sequences, enhancing tumour delineation across all sequences.

To assess reproducibility, reader 2 performed independent segmentations, blinded to reader 1’s results. For intrareader agreement, reader 1 re-labelled 10 cases after a 2-month interval. Additionally, reader 1 measured three-dimensional tumour diameters (*a*, *b*, *c*) on Gd-enhanced T1-weighted MR images pre- and post-chemotherapy, calculating tumour volume using the ellipsoid formula: *V* = *πabc*/6.

### Convolutional neural network architecture

**Fig. 2:**
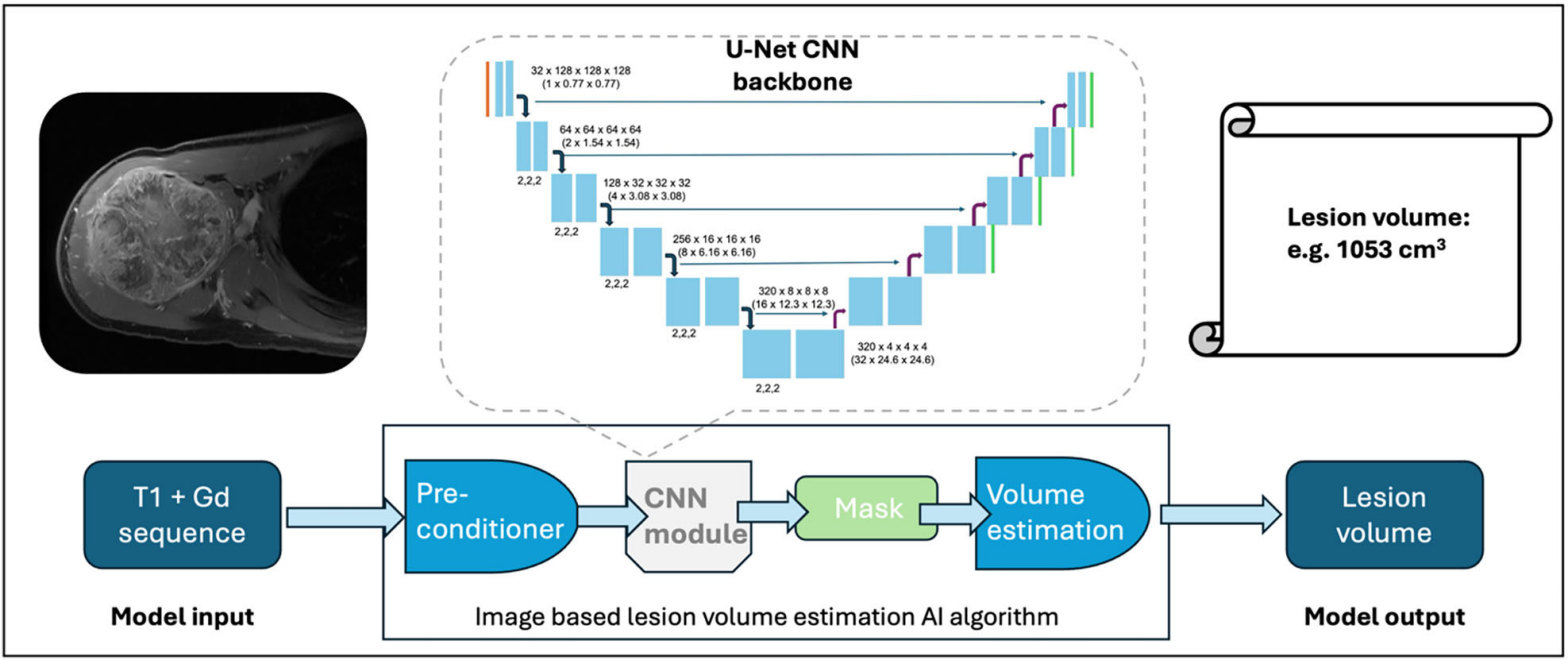
Osteosarcoma lesion volume estimation model. Contrast-enhanced T1-weighted MR images (T1+Gd) are first pre-processed to standardise image properties. A convolutional neural network (CNN) then performs automated 3D segmentation of the tumour, based on the approach of radiologist-delineated contours. The resulting lesion mask is converted into a volumetric measurement in mm³. The diagram illustrates the main processing steps of this pipeline

Our volume estimation algorithm processes contrast-enhanced T1-weighted MR images (input) to generate the volume of the detected osteosarcoma lesion (output) in mm^3^ (Fig. 2). The method applies a pre-conditioning step to standardise image properties, followed by automated 3D segmentation with a CNN trained on radiologist-delineated contours (7 years’ experience). The CNN was trained with a cross-entropy/Dice loss and Adam optimisation [13], using ½,½,½ subsampling and a 128 × 128 × 128 input patch size to balance geometric fidelity and dataset constraints. The model comprises 12 convolutional layers, ReLU activations, and 31.1 M parameters (118.69 MB). Training was performed on 162 MRI scans from 81 patients, with validation on 20 internal and 20 external scans (Centres A and B). External validation served as a robustness check, as these scans were acquired independently and with different equipment from the training data.

### Outcome prediction model

**Fig. 3:**
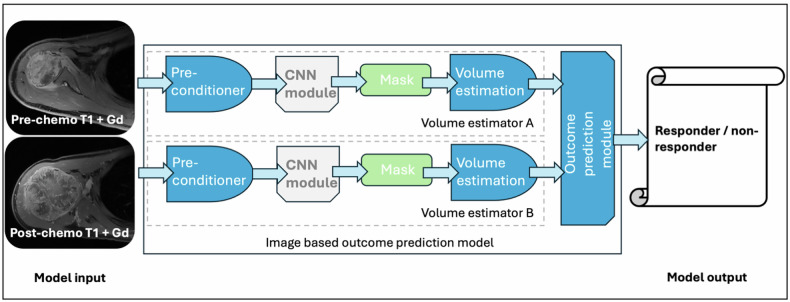
Outcome prediction model for treatment response in osteosarcoma. The diagram shows how pre- and post-chemotherapy contrast-enhanced T1-weighted MR images (T1+Gd) are each processed by a dedicated volume estimator. The resulting baseline and post-treatment volumes are used to calculate tumour volume change, which is input to a prediction module trained to classify patients as histological responders (≥ 90% necrosis) or non-responders

We developed an image-based outcome prediction model that incorporated our CNN-based volume estimator as a key sub-module and was designed to predict treatment response, defined as achieving ≥ 90% necrosis on histology (Fig. 3). Tumour volumes were calculated for both the pre-treatment baseline MRI scan and the post-induction MRI scan using separate instances of the volume estimation model. The change in tumour volume between the two scans was computed as:

$$\Delta {{{\rm{Tumour\; volume}}}}={{{{\rm{Volume}}}}}_{{{{\rm{Baseline}}}}}-{{{{\rm{Volume}}}}}_{{{{\rm{Post}}}}-{{{\rm{Induction}}}}}$$

This change served as the primary imaging biomarker for treatment response. The outcome prediction module applied logistic regression, trained with radiologist-labelled imaging features (baseline volume, post-induction volume, and Δ tumour volume) and outcome labels derived from histopathological necrosis measurements. Histopathology served as the gold standard, categorising patients as responders (≥ 90% necrosis) or non-responders (< 90% necrosis).

Training excluded all data used for validating the volume estimation model. The final architecture comprised three high-level blocks: two CNN-based volume estimators for the longitudinal MRI inputs, and an outcome prediction module. Model performance was evaluated against histological response classifications in terms of accuracy, sensitivity, and specificity.

### Statistical analysis

We used Python v3.10 (2021) and the following open-source libraries: NumPy v2.1.0 (2024), SciPy v1.13.2 (2023), Pandas v2.2.3 (2024), and Scikit-learn v1.5 (2024). AI segmentation performance was evaluated using the Dice coefficient, Hausdorff distance, and volume delta, presented in box plots. The Dice coefficient measured spatial overlap between CNN-predicted segmentation and the radiologist-defined ground truth at the voxel level. To assess agreement, Spearman’s correlation analysed AI- vs. human-derived tumour volumes, while Bland–Altman analysis quantified differences. Inter-observer and intra-observer variability in tumour volume measurements were assessed using the intraclass correlation coefficient (ICC) for consistency across different observers (inter-observer) and the same observer (intra-observer). The Dice coefficient further measured spatial overlap and delineation consistency. Model performance was evaluated using sensitivity, specificity, and accuracy. Sensitivity reflected the proportion of patients correctly identified with > 90% necrosis (true positives), while specificity measured correctly identified non-responders (true negatives). Diagnostic accuracy represented the overall proportion of correct predictions in the validation cohort. As this was a retrospective study, no prospective sample size estimation was performed. All eligible cases with available imaging and histopathology data were included, and a *p*-value < 0.05 indicated statistical significance for all analyses.

## Results

### CNN performance and validation

**Fig. 4:**
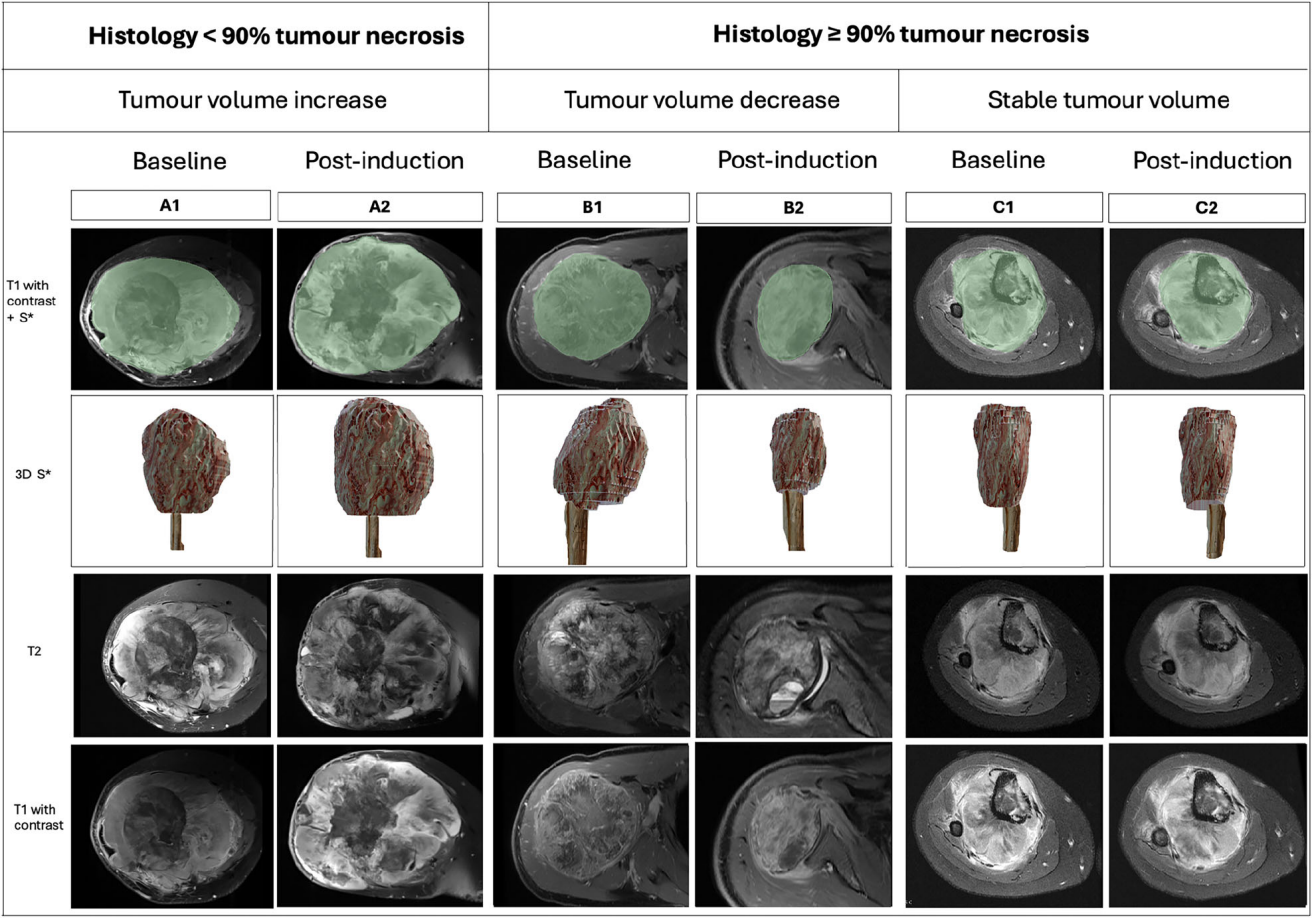
AI-derived tumour segmentations with corresponding MR images of osteosarcomas at baseline and post-induction chemotherapy. **A1**–**A2** Tumour without histological response (tumour necrosis < 90%) in an 11-year-old male, showing significant tumour growth after induction therapy. **B1**–**B2** Tumour with histological response (tumour necrosis ≥ 90%) in a 7-year-old female, demonstrating a noticeable decrease in tumour size post-therapy. **C1**–**C2** Tumour with histological response (tumour necrosis ≥ 90%) in a 14-year-old female, showing stable tumour volume after induction therapy. For each case, AI-derived tumour segmentations are overlaid on gadavist-enhanced T1-weighted MR images, with corresponding 3D tumour segmentations (3D S*) illustrating tumour volume changes between baseline and post-induction chemotherapy. MR images include a T2-weighted spin-echo sequence (TR = 3000 ms, TE = 100 ms, Flip Angle = 90°) and a gadavist-enhanced T1-weighted LAVA sequence (TR = 5 ms, TE = 2 ms, Flip Angle = 15°)

Figure 4 shows AI-derived tumour segmentations overlaid on representative MR images at baseline and post-chemotherapy, illustrating tumour volume changes over time. 3D segmentations (3D S)* provide a detailed visualisation of volumetric alterations. Tumours with < 90% necrosis exhibited significant growth post-therapy, whereas those with ≥ 90% necrosis showed size reduction or stability. These results demonstrate the model’s ability to accurately track tumour progression and treatment response through segmentation and volume estimation.

The volume estimation CNN achieved Dice coefficients of 0.9 (Centre A) and 0.8 (Centre B), with mean Hausdorff distances of 15 ± 19 mm and 14 ± 8 mm, respectively. Performance was evaluated on both internal and external validation sets.

**Fig. 5:**
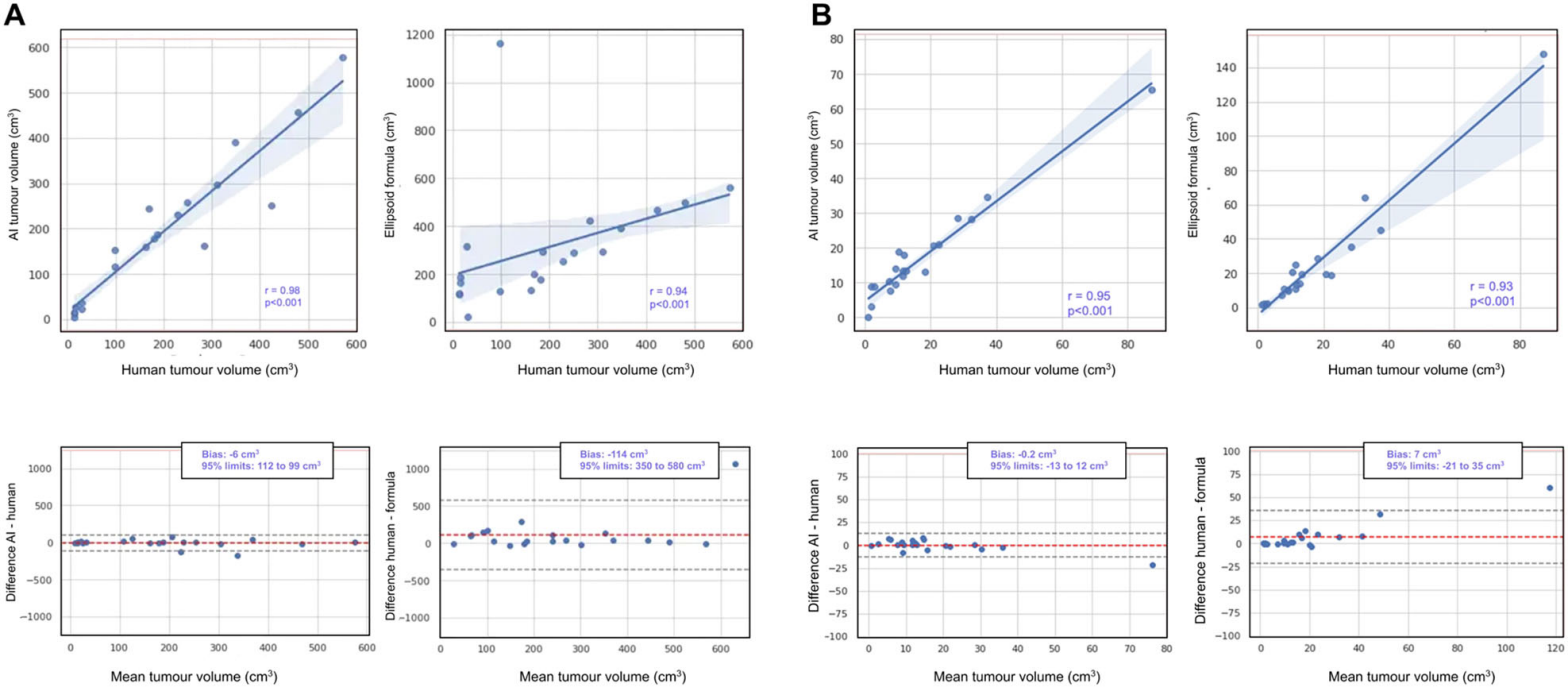
Comparison of AI-derived and human tumour volume measurements in internal and external validation datasets. **A** Spearman’s correlation and Bland–Altman analysis of AI-derived versus human tumour volume measurements for 10 subjects (20 MRI scans) in the internal validation dataset (Centre A). Scans were acquired pre- and post-chemotherapy. The Bland–Altman plot highlights agreement and potential bias between AI and human measurements. **B** Analysis of 10 subjects (20 MRI scans) in the external validation dataset (Centre B), with scans performed pre-chemotherapy and after at least two cycles of chemotherapy. Spearman’s correlation quantifies the relationship between AI-derived and human measurements, while the Bland–Altman plot illustrates agreement and systematic differences across datasets

In the internal validation set (Centre A), AI- and human-derived tumour volumes showed a strong correlation (*r* = 0.98, *p* < 0.001, Fig. 5A). Bland–Altman analysis indicated a mean bias of −6 mm³, with 95% limits ranging from −112 to +99 mm³, demonstrating minimal deviation. Human tumour volume calculations using the ellipsoid formula correlated significantly with human tumour volume measurements (*r* = 0.94, *p *< 0.001, Fig. 5A), while Bland–Altman analysis showed a mean bias of 115 mm³ with 95% limits from −351 to +580 mm³ (Fig. 5A).

In the external validation set (Centre B), AI- and human-derived tumour volumes also correlated strongly (*r* = 0.95, *p* < 0.001, Fig. 5B). Bland–Altman analysis revealed a mean bias of −0.2 mm³, with 95% limits from −13 to +12 mm³. Tumour volumes calculated using the ellipsoid formula correlated significantly with manual segmentation-based human measurements (*r *= 0.93, *p* < 0.001), with Bland–Altman analysis showing a mean bias of 7 mm³ and 95% limits from −21 to +35 mm³ (Fig. 5B).

### Repeatability of tumour volume measurements

Inter-observer variability between reader 1 (307 ± 595 mm³) and reader 2 (328 ± 761 mm³) showed moderate agreement (ICC = 0.7, *p* = 0.001) with a mean difference of 27 ± 497 mm³ (Fig. S1). Intra-observer agreement between initial (199 ± 86 mm³) and repeat measurements (187 ± 94 mm³) was high (ICC = 0.9, *p* = 0.001) with a mean difference of 6.9 ± 89 mm³ (Fig. S1).

### Correlation of AI-generated response stratifications with histopathological response

**Fig. 6:**
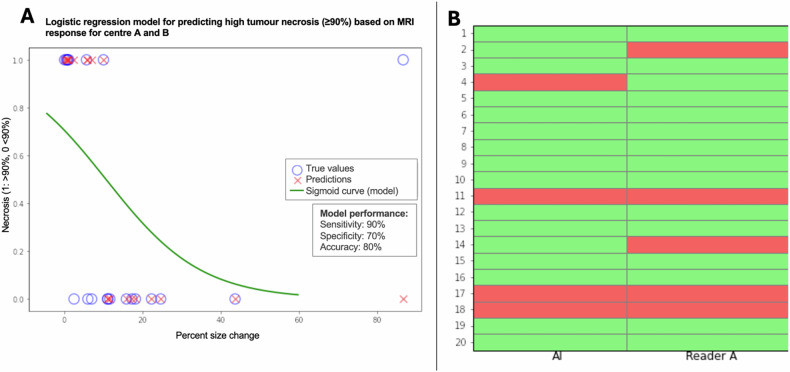
Outcome prediction using AI-based tumour volume estimation for osteosarcomas. **A** The performance of the AI-driven outcome prediction model applied to 20 osteosarcoma cases (Centres A and B combined) is illustrated. Tumour response was categorised as either shrinking or growing based on changes in tumour volume. The model predicts whether pathologic necrosis is ≥ 90%, achieving a prediction accuracy of 80%. Sensitivity and specificity were 90% and 70%, respectively. **B** Comparison of volumetric response classifications between the AI model and a human reader (Reader A) for each patient (rows 1–20). Green cells indicate correct predictions, while red cells denote misclassifications

Due to the small sample size, patients from Centre A and B were combined for response stratification. Histopathology showed > 90% necrosis in 12/20 osteosarcomas and < 90% necrosis in 8/20. Post-induction MRI scans revealed stable or decreased tumour volume in 12/20 cases and increased tumour volume in 8/20 cases. The outcome prediction model correctly classified 16/20 cases (Fig. 6), achieving 90% sensitivity, 70% specificity, and 80% diagnostic accuracy in differentiating responders from non-responders.

## Discussion

Our data showed that our CNN for measuring tumour volumes in osteosarcoma achieved strong segmentation performance, with Dice coefficients of 0.86 (Centre A) and 0.81 (Centre B), and precise Hausdorff distances. AI-derived tumour volumes also showed high correlation with human measurements. Additionally, a second CNN for predicting therapy response demonstrated 90% sensitivity, 70% specificity, and 80% accuracy.

Poor prognostic factors for patients with osteosarcoma include metastatic disease at baseline—which is associated with long-term survival rates below 25% [14], as well as less than 90% histological tumour necrosis after induction chemotherapy, and increasing tumour size [15, 16]. Shin et al found 3D MRI tumour volume changes in osteosarcoma significantly predicted outcomes after chemotherapy [17]. Therefore, patients exhibiting tumour growth during induction chemotherapy may benefit from earlier surgery and more aggressive chemotherapy regimens [18]. Traditionally, tumour volume changes have been estimated using the ellipsoid formula (*V* = *πabc*/6), which is based on the longest tumour diameters [19]. However, this approach may oversimplify tumour shape and response dynamics. Manual segmentation on CT/MRI, the reference standard for algorithm development, remains clinically impractical as this method is time-consuming, technically challenging, and requires specialised expertise [20**–**22]. The limitations of traditional volume estimation methods have led to the exploration of 3D volumetric approaches. Aghighi et al demonstrated that 3D tumour volume estimations outperformed 1D Response Evaluation Criteria in Solid Tumour (RECIST) and 2D WHO criteria in predicting Ewing sarcoma response. However, this method proved less accurate than true volumetric measurements [23]. Phillias et al demonstrated a direct correlation between increased tumour size and a higher risk of skeletal metastases in high-grade osteosarcoma, highlighting the prognostic importance of assessing tumour burden. [15]. In addition to volumetric parameters, treatment-related imaging changes have also been explored. Bishop et al reported an inverse correlation between the predictability of tumour necrosis and the intensity of therapy, suggesting that longer and more intensive treatment regimens reduce predictive accuracy [4]. Taken together, these findings suggest that changes in tumour volume may serve as an additional prognostic marker, offering valuable insights for risk stratification and guiding treatment adaptation. Kim et al found that tumour volume increase, measured via the ellipsoid formula, independently predicted shorter metastasis-free and overall survival (OS) in AJCC stage II osteosarcoma, while the adjusted necrosis rate also correlated with worse outcomes [24]. Similarly, Moon et al reported that 3D MRI-based tumour volume changes significantly predicted disease-free survival in high-grade osteosarcoma [25]. However, linear measurements remain limited by low reproducibility, with intra-observer variability of 6–14% and inter-observer variability up to 25%, as shown by Zhao et al [26].

AI-based segmentation improves efficiency and reproducibility over manual delineation. While few studies have focused on MRI-based osteosarcoma segmentation, Hasei et al developed an X-ray AI model (sensitivity: 95.5%, specificity: 96.2%, AUC: 0.99) [27], and Huang et al introduced a CT-based method (Dice: 87.8%) [20]. Ouyang et al proposed UATransNet, an MRI-based U-Net model incorporating self-aware attention (Dice: 0.9) [28], achieving segmentation accuracy comparable to our study. Beyond osteosarcoma, AI segmentation was successfully used with other neoplasms. Kawaguchi et al showed that AI-derived solid tumour volumes improved survival prediction in stage I lung adenocarcinoma (AUC: 0.8) [29], while deep learning models for meningiomas achieved segmentation accuracy comparable to human experts (Dice: 0.9) [10]. In NSCLC, 3D-Slicer-based AI contouring demonstrated stronger correlation with pathology and reduced inter-observer variability [30]. However, AI applications in paediatric bone tumours remain poorly characterised, largely due to challenges regarding the pathologies involved and limited data, with most recently published AI algorithms focusing on brain tumours [31]. To date, a few studies have investigated AI applications in paediatric lymphoma [32], neuroblastoma [33], Wilms tumour [34], and neuroendocrine tumours [35]. These efforts have primarily centred on image segmentation, demonstrating performance comparable to adult studies, while applications in response prediction or outcomes modelling remain largely unexplored.

Our second algorithm, also based on a CNN approach, predicted histopathologic response with 80% accuracy. While Teo et al conducted a similar study, they used a simpler classification model based on Fuzzy C-Means clustering and weighted majority voting on a small cohort (*n* = 10) [36]. Similarly, Bouhamama et al developed an MRI-based model for preoperative risk stratification in patients receiving neoadjuvant chemotherapy with an AUC of 0.95 [37]. However, their approach was radiomics-based and therefore difficult to directly compare to our CNN. Furthermore, our CNN-based model classified 16/20 cases correctly, slightly outperforming human measurements (15/20 cases), highlighting its potential clinical utility in therapy response prediction.

This study has several limitations. First, its retrospective design could introduce selection bias and limit generalisability. Second, the small sample size, especially in external validation, reduced its statistical power. The limited number of patients from the external centre may restrict the ability to fully assess model performance in heterogeneous clinical environments and across imaging platforms. As a result, the generalisability of the findings should be interpreted with caution. Third, CNN models were trained on MRI scans from two centres, which could limit performance across different imaging protocols. Fourth, manual segmentations as the reference standard are subject to variability. Fifth, the model evaluated treatment response based on histopathologic tumour necrosis rather than event-free or OS. While histologic response is a commonly used surrogate, it may not fully capture long-term clinical outcomes. Tumour volume shrinkage was used as the primary imaging biomarker, but in paediatric oncology, favourable responses can also involve architectural changes such as necrosis or cystic degeneration. Therefore, relying solely on volume may underestimate the true response to treatment. Future studies should explore additional imaging characteristics such as texture, internal composition, and functional parameters. Although the patient cohort was relatively small, it was nevertheless significant given the rarity of osteosarcoma in this population, and the distribution of good and poor responders was well-balanced. To improve robustness and enhanced clinical applicability, prospective, multicentre datasets with larger and more diverse patient populations, as well as the integration of additional outcome measures and clinical variables, are required.

In conclusion, this study presents a fully automated CNN for tumour volume estimation in osteosarcoma, achieving performance comparable to, and with a trend toward superiority over, conventional methodologies. Tumour volume changes quantified by the algorithm following induction chemotherapy were validated as predictors of histological tumour necrosis, highlighting its potential in prognostic assessments. These findings support the incorporation of AI-driven volumetric analysis into clinical workflows, with the potential to refine patient management and improve therapeutic outcomes in osteosarcoma.

## Supplementary information

Supplementary information

## Abbreviations

AUC: Area under the curve
CNN: Convolutional neural network
Gd: Gadolinium
ICC: Intraclass correlation coefficient
MAP: Neoadjuvant chemotherapy with high-dose methotrexate, doxorubicin, (cis)platin
MRI: Magnetic resonance imaging
OS: Overall survival
RECIST: Response evaluation criteria in solid tumours
ReLU: Rectified linear unit
WHO: World Health Organization
