# Supplementary material for: Deep learning for accurate tumour volume measurement and prediction of therapy response in paediatric osteosarcoma
Source: Eur Radiol. 2025 Nov 1;36(4):2458–68. doi: 10.1007/s00330-025-12115-w (PMC12694974; doi:10.1007/s00330-025-12115-w)
Supplement: Supplementary file 1 — Supplementary information [file 330_2025_12115_MOESM1_ESM.pdf]

Deep learning for accurate tumour volume measurement and prediction of therapy response in paediatric osteosarcoma

ELECTRONIC SUPPLEMENTARY MATERIAL

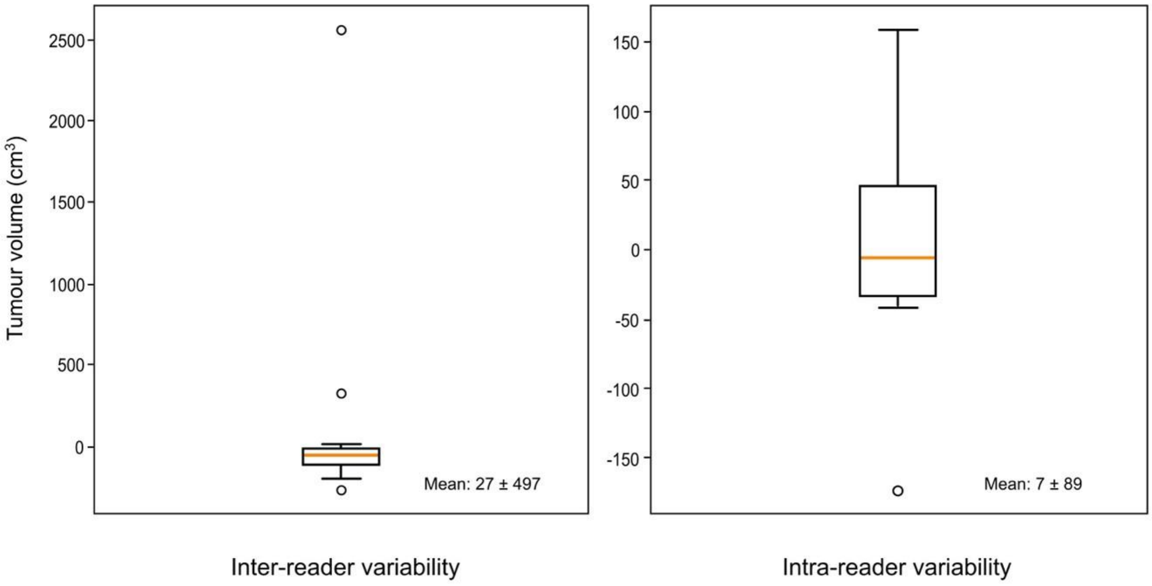

Figure S1: Inter- and intra-reader variability in tumor volume measurements. Box plots comparing inter-reader and intra-reader variability in tumor volume measurements, expressed in cubic centimeters (mm³). The left panel shows the inter-reader variability between two human observers, with a mean difference of 27 ± 497 cm³. The right panel illustrates the intra-reader variability for repeated measurements by a single observer, with a mean difference of 7 ± 89 cm³. The orange lines represent the median, the box spans the interquartile range, and the whiskers extend to 1.5 times the interquartile range. Outliers are plotted as individual points.
